# Editorial: Equity in type 1 diabetes technology and beyond: where are we in 2022?

**DOI:** 10.3389/fendo.2024.1400240

**Published:** 2024-03-26

**Authors:** Marie-Anne Burckhardt, Ananta Addala, Martin de Bock

**Affiliations:** ^1^ Pediatric Endocrinology and Diabetology, University Children’s Hospital, Basel, Switzerland; ^2^ Department of Clinical Research, University of Basel, Basel, Switzerland; ^3^ Division of Endocrinology, Department of Pediatrics, Stanford University, Stanford, CA, United States; ^4^ Stanford Diabetes Research Center, Stanford University, Stanford, CA, United States; ^5^ Department of Paediatrics, University of Otago, Christchurch, New Zealand

**Keywords:** type 1 diabetes, diabetes technology, equity, children and adolescents, disparities

The uptake of diabetes technology, such as continuous glucose monitoring (CGM), insulin pumps and automated insulin delivery, is rapidly increasing in people with type 1 diabetes (T1D), particularly in young people with T1D. Diabetes technology has the potential to be a game-changer, offering improved glycemic control and better psychosocial outcomes. However, it is crucial to acknowledge the existing disparities that hinder equitable access to these advancements. Diabetes technology uptake varies greatly by country, socioeconomic status, race, ethnicity, insurance coverage, and a myriad of social determinants of health (1–4). Inequity in access to technology is one of the key divers of disparities and may become an even more pronounced driver of inequities as the diabetes technology is evolving (5). It has been shown that diabetes technology use is lowest and HbA1c is highest in those from the low socioeconomic status groups (4). This Research Topic encloses high-quality manuscripts on the current topic of equity in T1D technology.

One of the major barriers to access to diabetes technology use is payer coverage (6). Lomax et al. compared two different funding models for T1D technology in Australia evaluating differences in technology use across socioeconomic groups. Nationally subsidized CGM use showed similar use across all groups except for the most disadvantaged group who had lower use. Current user pays model implemented for pump use in Australia revealed a pattern of increasing pump use with higher socioeconomic status group, with the lowest pump use in socioeconomically most disadvantaged people living in Australia.

To complement the user pays model in Australia and provide access to insulin pump therapy to people who do not have private health insurance to cover insulin pump therapy, alternative pathways such as government or hospital funded programs to access this technology are in place. Fu et al. investigated a sample of children who accessed insulin pump therapy through such subsidization program. These children were able to maintain healthier glycemia for two years. Families favored pumps as a management option. However, financial limitations persisted as a significant barrier to continue pump therapy. Taken together, these data from Lomax et al. and Fu et al. point to nationally subsidized T1D technology coverage as a strategy to bridge disparities.

Even if economic barriers such as funding are overcome, access to technology is not guaranteed. This may be due to social and health system disparities among others. For children, adolescents, and young adults, access to diabetes technology varies by migration background and gender differences in pump use exist (7). Auzanneau et al. showed that this is also the case in adults with T1D: higher age, male gender, and migration background are currently associated with lower use of diabetes technology in adults with T1D in Germany. The authors also explored area deprivation, defined as a relative lack of area-based resources, and showed a non-linear association, most likely due to correlations with other factors. Furthermore, cultural factors and technological literacy may play a crucial role in shaping the acceptance and utilization of T1D technology (8). Education and peer support programs play a pivotal role in ensuring that individuals with T1D can make informed decisions about their care. However, disparities in education and awareness persist, with some communities lacking the necessary information or workforce to understand and utilize advanced technologies.

Addressing the issue of equity in T1D technology requires effort from everyone involved in diabetes technology: users (individuals with T1D and clinicians), researchers, technology developers, and policymakers. An excellent setting to assess how consistently equity is discussed is an international diabetes technology meeting where experts from all these fields are present and groundbreaking research efforts are presented. Leadley et al. analyzed oral presentations of an internationally renowned diabetes technology conference and described what percentage of speakers discussed equity in their talks. They found that less than a quarter of presenters discussed equity. To ensure that diabetes technologies reduce disparity and improve outcomes, future speakers at diabetes and diabetes technology conferences should be supported to consider equity of diabetes care.

Equity in T1D technology is not limited to developed nations. Low- and middle-income countries face additional challenges, including limited healthcare infrastructure, lack of awareness, and financial constraints (1). Global initiatives, partnerships, and collaborations are essential to bring advanced diabetes technology to underserved populations worldwide. Achieving equity in T1D technology is not just a matter of scientific progress; it is a call for social responsibility and a commitment to ensuring that all individuals, regardless of socio-economic status, have equal access to life-changing innovations. We advocate for a collective effort from policymakers, healthcare providers, technology developers, and the community at large to bridge the divide.

## Author contributions

MAB: Conceptualization, Writing – original draft, Writing – review & editing. AA: Writing – review & editing. Md: Writing – review & editing.
